# Characterization of ZnO nanoparticles grown in presence of Folic acid template

**DOI:** 10.1186/1477-3155-10-29

**Published:** 2012-07-12

**Authors:** Sreetama Dutta, Bichitra N Ganguly

**Affiliations:** 1 Applied Nuclear Physics Division, Saha Institute of Nuclear Physics, Kolkata, 700064, India

**Keywords:** ZnO nanoparticles, Folic acid, Structural effects, Spectroscopic study, Charge transfer effects

## Abstract

**Background:**

ZnO nanoparticles (grown in the template of folic acid) are biologically useful, luminescent material. It can be used for multifunctional purposes, e.g., as biosensor, bioimaging, targeted drug delivery and as growth promoting medicine.

**Methods:**

Sol–gel chemical method was used to develop the uniform ZnO nanoparticles, in a folic acid template at room temperature and pH ~ 7.5. Agglomeration of the particles was prevented due to surface charge density of folic acid in the medium. ZnO nanoparticle was further characterized by different physical methods.

**Results:**

Nanocrystalline, wurtzite ZnO particles thus prepared show interesting structural as well as band gap properties due to capping with folic acid.

**Conclusions:**

A rapid, easy and chemical preparative method for the growth of ZnO nanoparticles with important surface physical properties is discussed. Emphatically, after capping with folic acid, its photoluminescence properties are in the visible region. Therefore, the same can be used for monitoring local environmental properties of biosystems.

## Introduction

Nanometer size multifunctional materials are gearing the biological fields in various ways [1]. One of the promising nontoxic and biocompatible semiconductor material is Zinc Oxide (ZnO), which has received extensive application due to its exceptional electrical and optical characteristics [2] in fabricating nanoscaled electronic and optoelectronic devices. ZnO is a kind of wide band gap (3.37 eV) semiconductor with large exciton binding energy (60 meV) [2]. In comparison to other wide band-gap semiconductors, ZnO possesses higher quantum efficiency [3] and higher exciton energy [4,5]**.** Also, ZnO is a biofriendly oxide semiconductor and an inexpensive luminescent material. Owing to the properties stated above, it is expected to have a wide range of applications in room temperature ultraviolet (UV) lasing [6], biosensors [7], bioimaging [8], drug delivery [9] and piezoelectric transducers [10]. In general, ZnO is considered “generally recognized as safe” (GRAS) [11] but ZnO nanoparticle system may be toxic. ZnO nanosystem may be of important relevance in the context of nanomedicine, where targeted treatment of biological systems at molecular level is a necessity [12]**.**

Recently, there are several physical or chemical synthetic methods of preparing ZnO, such as thermal evaporation [13], pulsed laser deposition (PLD) [14], ion implantation [15], reactive electron beam evaporation [16], thermal decomposition [17] and sol–gel technique [18-22]. To obtain ZnO nanoparticle, we choose sol–gel method because of its simplicity, which offers a possibility of large-area yield at low cost.

**Figure 1:**
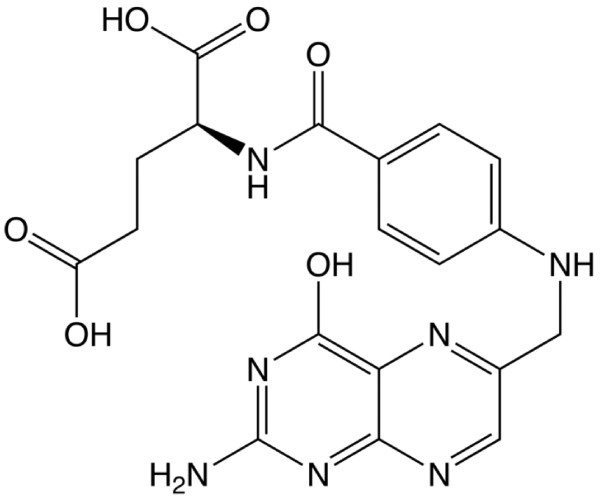
Molecular structure of folic acid.

In the present study, nano-sized ZnO sample has been prepared by chemical synthesis in presence of surface active biological substance, such as folic acid. Folic acid [23,24] is a member of the Vitamin B family and is necessary for the healthy function of a variety of bodily processes. The structural aspect of the folic acid is shown in Figure 1. Folic acid is sparingly soluble in pure water, but is well dispersed under physiological pH ~ 7.5. Folic acid being a multi dentate ligand, helps in controlling ZnO nanoparticle size through its surface charge density [23]. Also, folic acid has a natural affinity towards Folate receptor protein, which is over expressed by a number of tumor cells [25]. Since ZnO nanoparticles are cytotoxic and can combat the growth of tumor cells, it is envisaged that such a capping would help in targeting tumor cells. In this article further, the structural effects and the influence of folic acid are discussed in detail with the help of physical methods and spectroscopic tools. It is envisioned that the simple preparative scheme of the compound and the physical characteristics as shown in this article, would find its vital pathway in biotechnological applications and as well as optoelectronic device forming material.

## Materials and methods

### Chemical method

#### Chemical Synthesis of pure Zinc oxide (ZnO)

**Figure 2:**
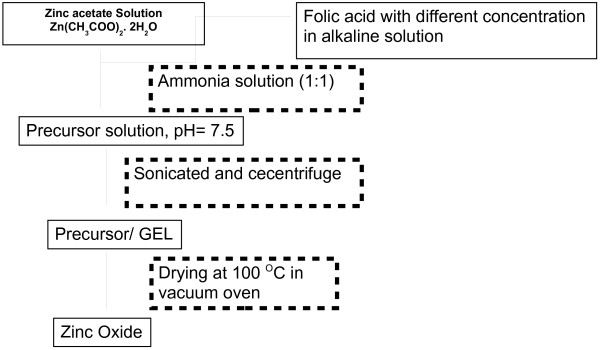
Flow chart of chemical preparation of ZnO nanoparticle in presence of folic acid template.

ZnO nanoparticles were prepared by the sol–gel technique (shown in Figure 2) from the zinc acetate (Zn(CH_3_COO)_2_. 2H_2_O, extra pure AR, grade material, from SRL, India). Desired weight of zinc acetate was dissolved in triple distilled water (TDW) and (1:1/vol) ammonia solution (Merck India) was added to this solution drop by drop, maintaining pH ~ 7.5; initially zinc precipitated as zinc hydroxide. After centrifugation, the precipitate has been collected and re-dispersed into TDW for removing of excess ions. Finally, the precipitate was recollected and dried at 100°C to get ZnO.

#### a) *ZnO grown under Folic acid template*

Folic acid (M.F.: C_19_H_19_N_7_O_6_, procured from Sigma. life Science), was dissolved in mildly alkaline TDW [26] at different percentage concentrations. Folic acid solution of desired dilution was added to zinc acetate solution and the final pH was adjusted to 7.5. The samples are denoted as Z_0.2_, Z_0.5_, Z_1.0_, Z_1.3_, Z_2.0_, Z_3.0_ and Z_4.8_. The suffix (Z_x_) represents the percentage concentration (weight/volume) of folic acid solution. After centrifugation, the precipitate was collected and re-dispersed into TDW for removal of excess ions. Finally, the precipitates were recollected and dried at 100°C. The schematic representation of the chemical synthesis is given in Figure 2. The prepared samples have been characterized by various physical techniques as given in the following classified sections.

### Physical methods of characterization of the ZnO nanoparticles

#### X-ray diffraction (XRD) measurements

**Figure 3:**
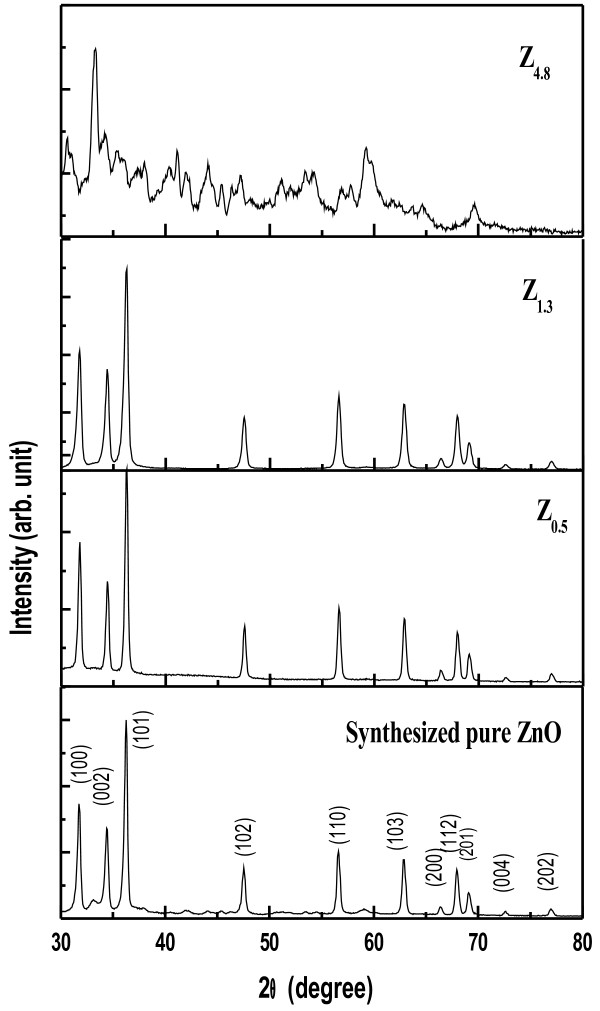
X-ray diffraction patterns with (hkl) values of as-grown ZnO nanoparticles by sol–gel method, where folic acid concentration (weight/volume) has been (a) 0%, (b) 0.5%, (c) 1.3%, (d) 4.8%.

The phase structures of the samples were identified by X-ray diffraction technique using Seifert XDAL 3000 diffractometer with CuKα radiation (wavelength of the radiation, k = 1.54 Å). The data have been collected in the range (2θ) 30° –80° with a step size of 0.06°. Si has been used as external standard to deconvolute the contribution of instrumental broadening [27]. The XRD pattern has been shown in Figure 3.

The grain sizes of the synthesized samples have been calculated using Scherrer formula [27]:

(1) $${\text{D}}_{\text{hkl}}=\frac{\text{K}\lambda }{\beta_{\text{P}}\text{cos}\theta }$$

where, D_hkl_ is the average grain size, K the shape factor (taken as 0.9), λ is the X-ray wavelength, β_P_ is the full width at half maximum (FWHM) intensity (here 101 peak of the ZnO spectrum fitted with a Gaussian, for precision measurement) and θ is the Bragg angle.

The nano crystalline material usually suffers from structural strain as the grain interior is relatively defect free but the grain boundary consists of high-density defect clusters [28,29]. Thus, the strain in the lattice has been estimated through constructing Williamson–Hall (W–H) plot, with different Bragg peaks [30] taken in to consideration, such as:

(2) $$\beta \text{cos}\theta =\text{K}\lambda /{\text{D}}_{\text{hkl}}+2ɛ\text{sin}\theta$$

where, ϵ is the micro strain parameter.

Also, an estimation of the lattice parameters has been made by using FullProf program [31].

#### Transmission Electron Microscopic (TEM) study

The morphology of the synthesized product were characterized by transmission electron microscopy, TEM (Tecnai S-twin, FEI) using an accelerating voltage of 200 kV, having a resolution of ~ 1 Å. For this analysis, the ZnO sample has been dispersed in TDW through a probe sonicator; a drop of the same was placed onto a carbon coated copper grid and dried at room temperature. Furthermore, selected area electron diffraction (SAED) patterns are recorded to determine the growth orientation of the synthesized ZnO.

#### Spectroscopic Measurements

##### *i) Fourier transmission infrared (FT-IR) spectra*

Fourier transmission infrared (FT-IR) spectra of the powders (as pellets in KBr, without moisture) were recorded using a Fourier transform infrared spectrometer (Perkin Elmer FTIR system; Spectrum GX) in the range of 400–6000 cm^-1^ with a resolution of 0.2 cm^-1^.

##### *ii) UV –Vis Spectroscopic measurements*

The optical absorption spectra were measured in the range of 250–800 nm using a UV–VIS-NIR scanning spectrometer (Lamda 750, Perkin Elmer).

##### *iii) Room temperature Photoluminescence (PL) Spectroscopy*

Room temperature Photoluminescence (PL) measurement was carried out by a laser induced luminescence spectrometer (model IK3102R-G), the excitation source at room temperature being 325 nm line from a He-Cd laser.

## Results and discussions

### X-ray Diffraction (XRD) study

**Figure 4:**
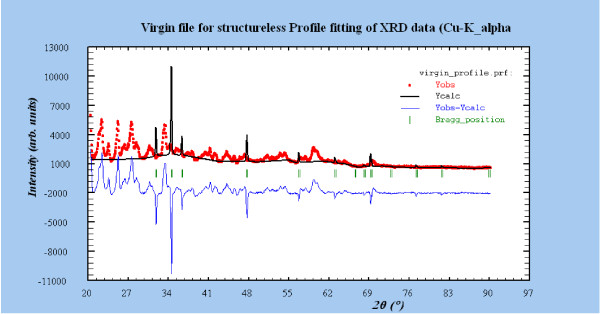
**XRD peak fitting for Z**_**4.8**_**sample using FullProf programming, notice Bragg position for crystallinity assessment.**

XRD results give us the characteristic diffraction pattern of the crystallites under the particular configuration, through a Bragg angle. Figure 3 shows the XRD patterns of the synthesized ZnO powder samples in presence of folic acid template. The appearance of characteristic diffraction peaks for pure ZnO sample corresponding to (1 0 0), (0 0 2), (1 0 1), (1 0 2), (1 1 0), (1 0 3) and (1 1 2) planes is in good agreement with the standard XRD peaks of crystalline bulk ZnO with hexagonal wurtzite structure [JCPDS card No. 36–1451, a = 3.2501 Å, c = 5.2071 Å, space group: P_6_3mc (1 8 6)], except for Z_4.8_ sample, where partial crystallinity has been found. No characteristic peaks from the intermediates such as Zn(OH)_2_ can be detected in the samples stated above. The amorphous nature in Z_4.8_ sample is only due to the ensconced folic acid molecules due to its high concentration [32]. All the XRD data of the samples have been analyzed by FullProf programming as shown in Table 1. In order to have a clear idea of the partial crystallinity observed in the case of Z_4.8_ sample, the analysis (through FullProf programming) has been clearly shown in Figure 4 and Table 2. The analysis depicts that partial crystallinity of ZnO remains, despite the strong folic acid influence in the medium. The lattice parameters given in Table 1 for the synthesized ZnO samples are in accordance with standard data of ZnO wurtzite structure except for Z_1.0_ sample. The deviation here is ~ 11% in a-parameter and ~25% in c-parameter. This could be due to the structural transition in the crystallite, which we discuss later in detail.

**Table 1:** Lattice constant calculated from Fullprof programming

**Sample**	**a (Å)**	**b (Å)**	**c (Å)**
Pure ZnO	3.249105	3.249105	5.203271
Z_0.2_	3.249067	3.249067	5.201507
Z_0.5_	3.232881	3.232881	5.188294
Z_1.0_	3.134176	3.134176	4.959011
Z_1.3_	3.248979	3.248979	5.202200
Z_2.0_	3.232689	3.232689	5.195967
Z_3.0_	3.236962	3.236962	5.206523
Z_4.8_	3.242703	3.242703	5.194723

**Table 2:** **Miller indices (hkl) and corresponding peak position for Z**_**4.8**_**sample**

**h k l**	**peak position**
1 0 0	31.8360
0 0 2	34.5018
1 0 1	36.3329
1 0 2	47.6491
1 1 0	56.7232
1 0 3	63.0119
2 0 0	66.5315
1 1 2	68.1121
2 0 1	69.2511
0 0 4	72.7565
2 0 2	77.1527

**Figure 5:**
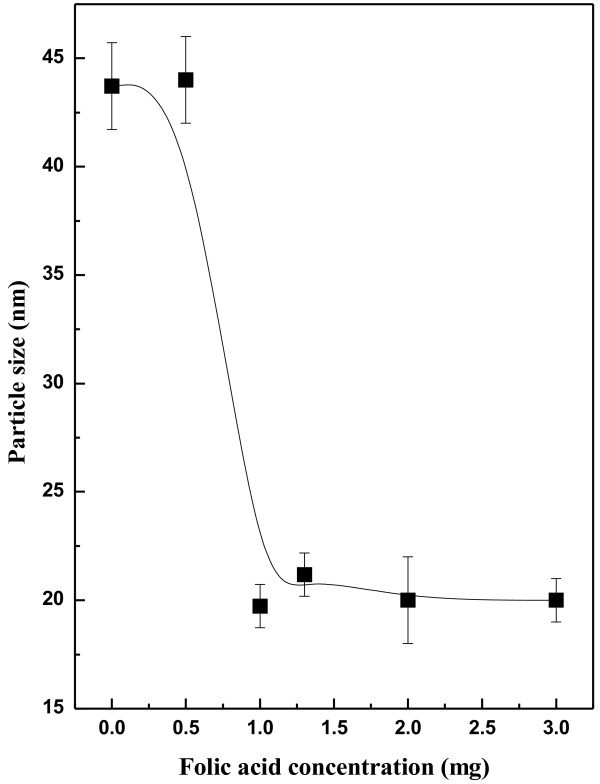
Relationship between ZnO grain size development and folic acid concentration (weight/volume), added during sol–gel preparation method.

Further, the average grain sizes of the ZnO samples were estimated from X-ray line broadening using Scherrer’s equation [27]. The particle size of pure ZnO is 41 nm, whereas it decreases to 20 nm with increase in folic acid concentration, shown in Figure 5. A sharp decrease in grain size (grain size ~ 18 nm) for Z_1_ sample has been shown in the results. The use of folic acid template has been effective after a certain concentration in controlling the Ostwald ripening [33] process in the growth rate of the crystallites. From the size effect of the ZnO shown the agglomeration number of the molecules in the case of each samples can be explained through simple relationship (assuming the small crystallites are roughly spherical for a minimal surface to volume ratio):

(3) $$\text{n}=4/3({\text{πr}}^{3}\text{p})\left({\text{N}}_{\text{A}}/\text{M}\right)$$

where, density of ZnO (ρ) = 5.606 gm/cm^3^, N_A_ is the Avogadro’s number, molecular weight (M) = 81.389 gm/mole.

**Figure 6:**
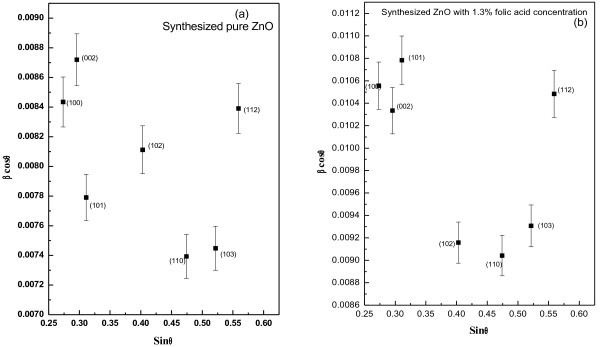
**Williamson–Hall plots for (a) pure ZnO and (b) Z**_**1.3**_**samples, for structural strain analysis.** Error bar indicates the structural broadening of particular peak.

It has been noticed from the results (Table 3), shown that the grain size decreases with folic acid concentration with a consequent decrease in agglomeration number of ZnO crystallites. One can estimate the strain in ZnO structure due to its size effect (grown in the presence of folic acid) by W-H plot, shown in Figure 6. Considerable anisotropy in structure has been noticed since unambiguous linear plot of the strain from all Bragg angles were not possible, the reason possibly lies with the surface effect of the crystallites. Although the results have been shown for only two representative samples [for pure ZnO (~ 21 nm) and Z_1.3_ samples (~ 29 nm)] but the trend has been maintained in case of all the samples.

**Table 3:** Average grain size, agglomeration number (n), surface to volume ratio of the crystallites and the molecular organization of ZnO crystallites grown without and with folic acid template

**Avg. grain size**	**n**	**Surface/volume**	**No. of molecules in the surface**
40 nm (pure ZnO)	4x10^24^	0.1	4x10^23^
20 nm	2x10^24^	0.3	6x10^23^
(with 3% folic acid concentration)			

### Morphological Investigation by TEM

**Figure 7:**
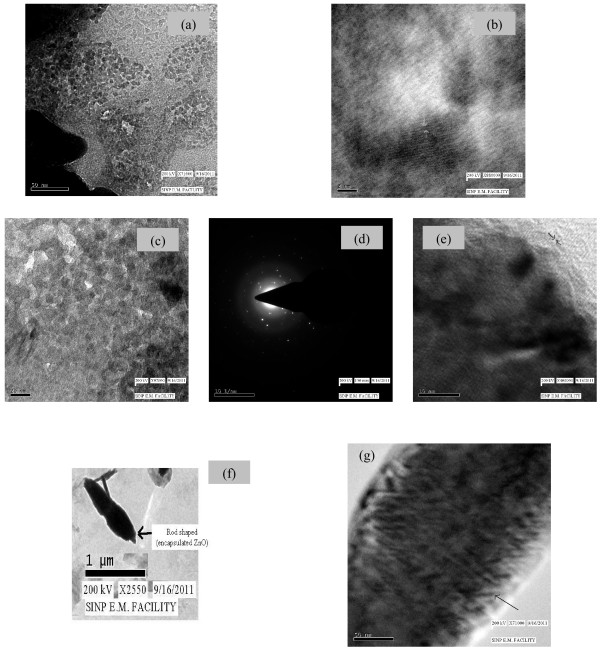
**TEM images for structural morphology of (a) pure ZnO sample and (b) the corresponding fringe patterns; (c) Z**_**1.3**_**sample and the corresponding (d) SAED and (e) fringe pattern; (f) Z**_**4.8**_**sample and (g) the corresponding fringe pattern of rod-like structure.**

Typical TEM images of different ZnO samples grown under folic acid template (pure, Z_1.3_, Z_4.8_) has been shown in Figure 7. TEM analysis was carried out for the determination of morphology, size and crystalline nature of the synthesized ZnO crystals. It can be estimated that the average size of ZnO lies nearly around 10 nm (from Figure 7(a)), which appears lower than the estimated results from Scherrer analysis. The fringes (shown in Figure 7(b)) in the microscopic analysis depicts the crystallinity of the selected zone in pure ZnO sample. Similarly, the wurtzite pattern has been shown in Z_1.3_ sample, shown in Figure 7(c) but with in the emblem of folic acid. Changes in the structure and size due to encapsulation by folic acid are quite evident from the Figure 7(c). However, the crystallinity pattern is maintained as shown in the subsequent results, in Figure 7(d) and (e). With increase in folic acid concentration, such as Z_4.8_ sample, a complete change in morphological structure has been found (shown in Figure 7(f)). The organization of nano-rods is evident. Further, the electron beam was focused on the nano-rods, fringe structure was repeatedly observed, as shown in Figure 7(g). Thus, we find at higher concentration of folic acid, although the ZnO granules are organized into nano-rod like structure yet a part of intrinsic crystallinity is retained.

However, the size estimate of nanoparticles from Scherrer method differs considerably, but it may be a rough estimate from the D_hkl_ values in the Scherrer’s formula. Both TEM and XRD method justifies the crystalline nature of the nanoparticles.

### Spectroscopic study

#### FTIR investigation

**Figure 8:**
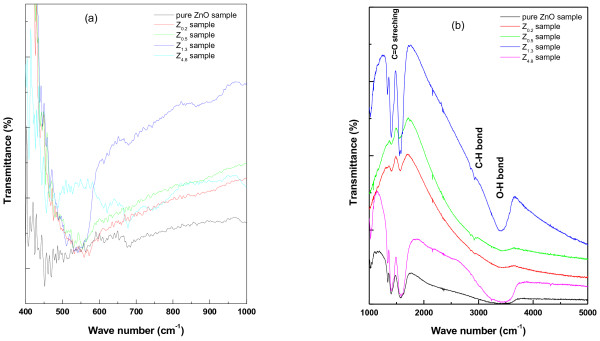
FTIR spectra of ZnO nanoparticles grown under the template of folic acid solution in (a) lower wave number and (b) higher wave number range (to show the residual effect of folic acid).

The structural analysis of wurtzite ZnO was further supported through FTIR investigation, shown in Figure 8. Figure 8(a) corresponds to the wurtzite oxide stretching frequencies of ZnO. The main absorption bands at ~ 450–500 cm^-1^(~ 4473x10^-10^- 4970 x10^-10^ joule), which is the stretching mode of ZnO, was considered [34-36]. In this context, it is observed that there is an influence of folic acid as the stretching modes have been shifted to higher energy states (with decrease in size of nanocrystallites). The effect of folic acid concentration on the starching frequencies of ZnO can be sensed through the calculation of the force constant

(4) $$\text{v}=1/2\text{πc }{\left[\text{K}/\text{μ}\right]}^{1/2}$$

and is tabulated as under Table 4[37]. These shifts may be related to change in bond length of Zn-O in the nanoparticles [37]. Further the FTIR spectra show absorption bands corresponding to the residual functional groups of folic acid template, as shown in Figure 8(b) and Table 5. Thus, it can be inferred that the influence of the template exists and ZnO electronic environment has been modified.

**Table 4:** IR frequency shift of Zn-O stretching frequency under the influence of folic acid

**Sample**	**IR frequency (cm**^**-1**^)
Pure ZnO	451
Z_0.2_	455
Z_0.5_	455
Z_1.3_	456
Z_4.8_	457

**Table 5:** FTIR absorption frequencies for residual groups of folic acid

**Wave number (cm**^**-1**^)	**Absorption band**
~ 3400	O-H mode
~ 2900	C-H mode
~ 1600	Symmetric C = O stretching mode
~ 1380	asymmetric C = O stretching mode

### UV–vis spectrum analysis

**Figure 9:**
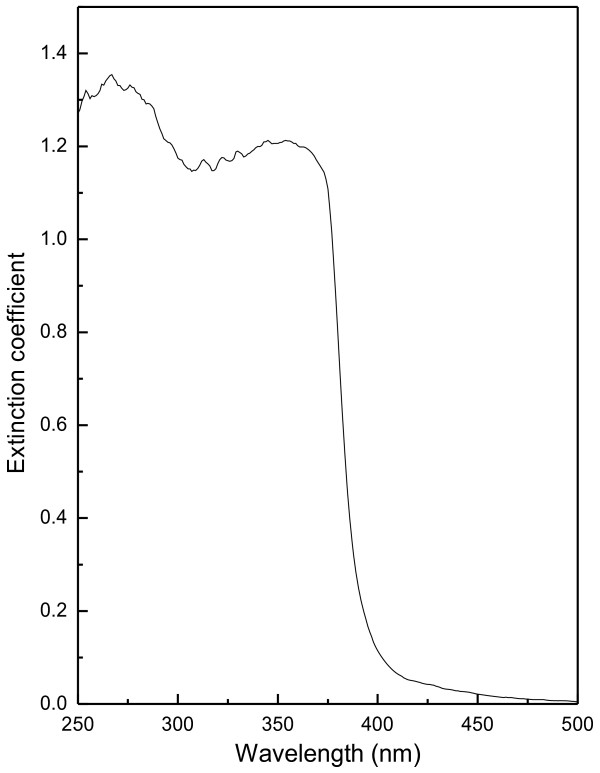
Optical absorbance spectra for pure ZnO (a representative spectrum in the range of 250–500 nm).

**Figure 10:**
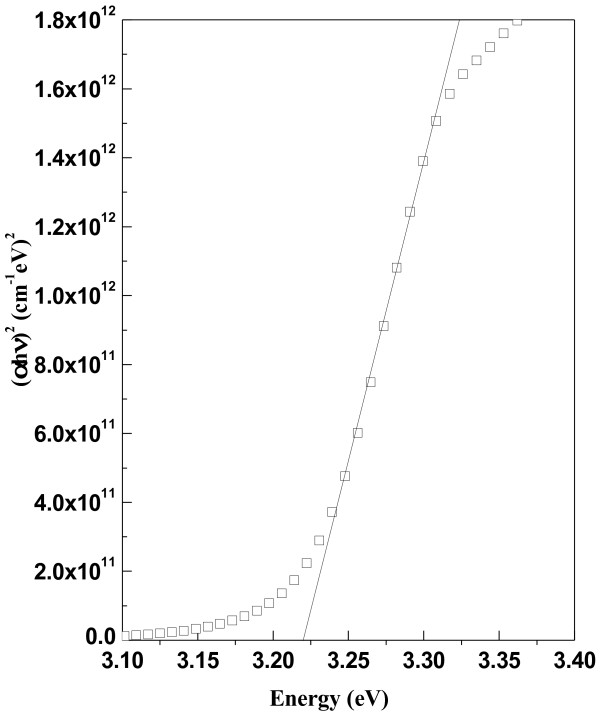
**Plots of (αhν)**^**2**^**vs photon energy for ZnO sample.**

**Figure 11:**
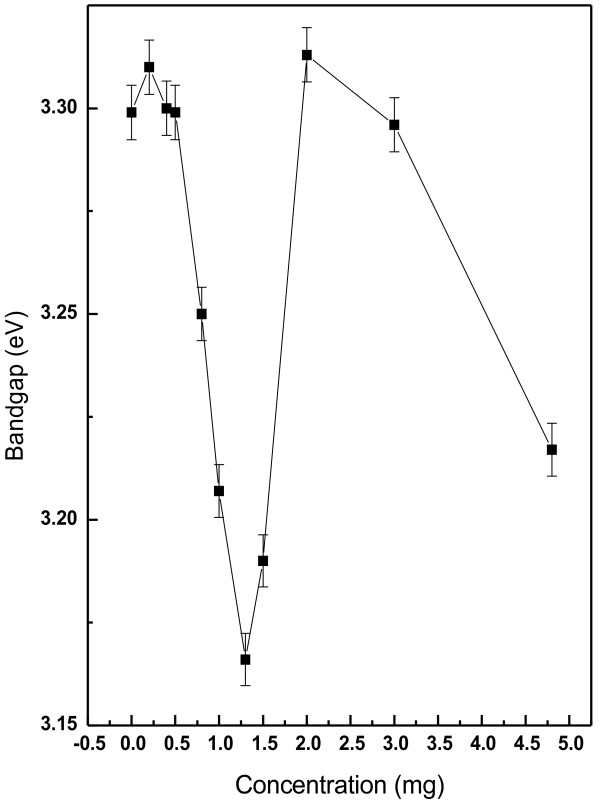
Dependence of the band gap (Eg) of all samples as a function of folic acid concentration.

The electronic absorption spectrum of ZnO samples in the UV–vis range enables to characterize the absorption edge related to semiconductor band structure. Figure 9 shows the UV–visible spectra of ZnO nanoparticles synthesized under folic acid template. The spectral absorption coefficient α(λ) has been evaluated [38] from the measured spectral extinction coefficient, *k(λ)*, using the following expression:

(5) $$\alpha \left(\lambda \right)=4\pi \text{k}\left(\lambda \right)/\lambda$$

where λ is the wavelength of the absorbed photon. The optical band gap (Eg) of the samples have been estimated from the well known expression [39] for direct transition, by fitting experimental absorption data with the equation

(6) $$\alpha \text{E}=\text{A}{\left(\text{E}-\text{Eg}\right)}^{\text{n}}$$

where E (=hc/λ) is the photon energy and *A* is a constant and ‘n’ depends on the kind of optical transition that prevails. Specifically, with n =1/2, a good linearity has been observed for the direct allowed transition, the most preferable one in the system studied here. Standard extrapolation of absorption onset (as shown in Figure 10) [39] to *αE* = 0 (where *E* = *E*g) has been made for each samples. The band gap of pure ZnO is ~ 3.3 eV, which is good agreement with other studies [40]. Overall decreasing nature of optical band gap (from 3.30 eV to 3.22 eV) has been observed in Figure 11, except for Z_1.0_ and Z_1.3_ samples, which lies in the structural transition zone. This effect is in corroboration with the XRD results, as referred in Figure 5. The red shift of the band gap due to systematic increase of folic acid concentration in the medium (at the sample preparation state) could be attributed to some defect state between valence band and conduction band (O_2p_ → Zn_3d_) [41]. The overall effect of this red shift of the band gap energy due to increase in folic acid concentration also relates to structural morphologies, particle size and surface microstructures [40]. However, a structural transition around Z_1_ and Z_1.3_ is evident from the result of XRD study (Figure 5) and optical absorption study (Figure 11). It would be worthwhile perhaps to recall the effect of subtle electronic environmental changes from IR spectroscopic data that conforms to this kind of structural changes.

### Photoluminescence spectroscopy (PL)

**Figure 12:**
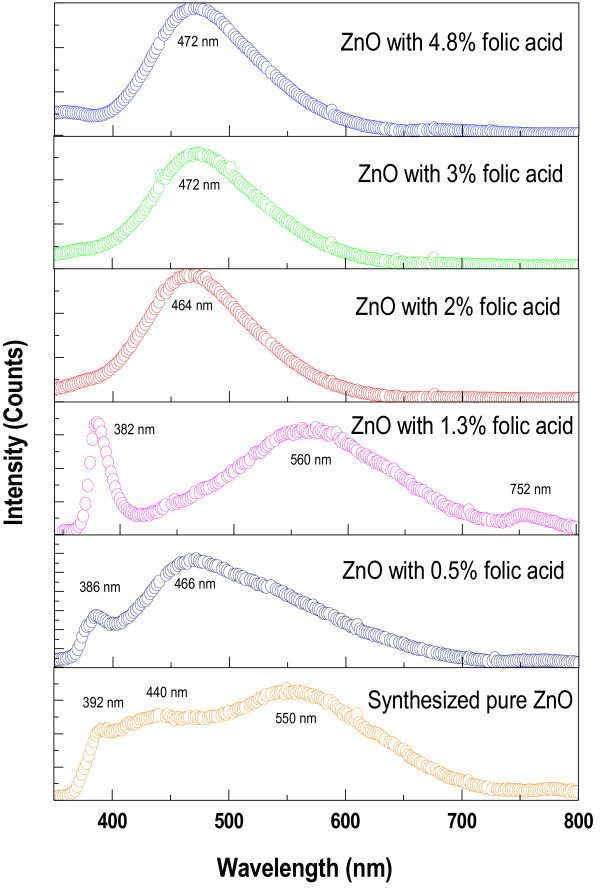
**Photoluminescence spectra of (a) pure ZnO, (b) Z**_**0.5**_**, (c) Z**_**1.3**_**, (d) Z**_**2.0**_**, (e) Z**_**3.0**_**, (f) Z**_**4.8**_**samples.**

The room-temperature PL spectroscopic study enables to determine the electronic energy levels from where emission is particularly observed which in turn helps to corroborate the band structure of the ZnO nanoparticles [42]. The results of PL spectrum of ZnO nanoparticles are presented in Figure 12. The pure ZnO spectrum shows a broad emission spectrum covering from near UV to whole of the visible region. With introduction of very low concentration of folic acid in the medium (Z_0.5_), a double peak emission spectrum becomes existent. This double peak structure is very prominent at Z_1.3_ sample, which is also the region exactly where a structural transition was observed with the size effect (vide Figures 5 and 11, band gap ~3.17 eV). The ultraviolet emission peak (UV) corresponds to an exciton emission band, whereas the visible peak is believed to be due to an electronic transition from a level close to the conduction band edge to a defect-associated trap state, such as an oxygen vacancy [43,44]. The UV emission is known as a near-band-edge (NBE) emission, originating from the recombination of free exciton through an exciton–exciton collision process [45].

In pure ZnO spectrum, a weak emission peak at 440 nm (blue emission) has been observed due to surface defect in ZnO, mainly due to Zn vacancy and broad green emission band (~ 550 nm), known as a deep level emission, relates to the deep-level defect states [46]. Singly ionized oxygen vacancy is responsible for this green emission in the ZnO [47]. It results from the recombination of the photo-generated hole with an electron, occupying the oxygen vacancy and interstitials of zinc.

In all samples, green light emission is most prominent. With increase in folic acid concentration in the medium (above 1.3%) a dramatic change in emission spectrum is observed. The spectrum now shows the emission peak only ~ 464 nm (for Z_2.0_) to 472 nm (Z_3.0_ and Z_4.8_) (with the blue shift of single emission peak), which is a signature of charge transfer reaction [48]. This clear transition is in corroborative confirmation of the effect of folic acid concentration on ZnO particle size and band gap properties shown by Figures 5 and 11. ZnO is now virtually ensconced structure with folic acid and the effect is drastic. The effect is also evident from FTIR (Tables 4 and 5) and TEM (Figure 7) study. The surface defects of ZnO are in the proximity of the functional groups of folic acid. Therefore, charge transfer effect becomes prominent and viable.

However, the physical mechanism behind visible light emission in ZnO is claimed by different authors in different ways and is still under controversy [49-52]. Therefore, it is important to investigate the luminescent mechanism caused by the defects of ZnO thin films, since they are the key factors for obtaining the visible luminescence. In our case, we find that the ZnO nanoparticle size decreases under influence of folic acid, there is a structural transition and finally the nano rod like structure is formed under the strong influence of folic acid. As a consequence to this the emission spectrum has shown the pronounced green light emission, which is conferred to photon induced charge transfer transition state.

## Conclusions

Influence of folic acid in controlling the structural effects of ZnO nanoparticle under physiological conditions of temperature and pH has been studied as a novel method. The physical investigations with XRD, TEM and spectroscopic tools have been carried out in order to understand the interesting structural changes involved in the system which may find important biomedical applications. Photo induced charge transfer due to folic acid ensconced ZnO nanosystem is particularly a noticeable effect as seen from our results.

## Competing interest

The authors declare that they have no competing interests.

## Author’s contribution

SD performed all the experimental works which was off-course verified by BNG. BNG organized the research works and given the research directives. All authors read and approve the final manuscript.
